# Alternative mechanisms for delivery of medication in South Africa: A scoping review

**DOI:** 10.4102/safp.v63i1.5274

**Published:** 2021-08-24

**Authors:** Robert Mash, Carmen Christian, Ruvimbo V. Chigwanda

**Affiliations:** 1 Division of Family Medicine and Primary Care, Faculty of Medicine and Health Sciences, Stellenbosch University, Cape Town, South Africa; 2 Department of Economics, Faculty of Economics and Management Sciences, University of the Western Cape, Bellville, South Africa; 3 Division of Social and Behavioural Sciences, School of Public Health and Family Medicine, University of Cape Town, Cape Town, South Africa

**Keywords:** primary health care, primary care, medication systems, adherence clubs, home delivery, alternative pick-up-points

## Abstract

**Background:**

The number of people in South Africa with chronic conditions is a challenge to the health system. In response to the coronavirus infection, health services in Cape Town introduced home delivery of medication by community health workers. In planning for the future, they requested a scoping review of alternative mechanisms for delivery of medication to patients in primary health care in South Africa.

**Methods:**

Databases were systematically searched using a comprehensive search strategy to identify studies from the last 10 years. A methodological guideline for conducting scoping reviews was followed. A standardised template was used to extract data and compare study characteristics and findings. Data was analysed both quantitatively and qualitatively.

**Results:**

A total of 4253 publications were identified and 26 included. Most publications were from the last 5 years (*n* = 21), research (*n* = 24), Western Cape (*n* = 15) and focused on adherence clubs (*n* = 17), alternative pick-up-points (*n* = 14), home delivery (*n* = 5) and HIV (*n* = 17). The majority of alternative mechanisms were supported by a centralised dispensing and packaging system. New technology such as smart lockers and automated pharmacy dispensing units have been piloted. Patients benefited from these alternatives and had improved adherence. Available evidence suggests alternative mechanisms were cheaper and more beneficial than attending the facility to collect medication.

**Conclusion:**

A mix of options tailored to the local context and patient choice that can be adequately managed by the system would be ideal. More economic evaluations are required of the alternatives, particularly before going to scale and for newer technology.

## Introduction

The South African health system is challenged by a number of colliding epidemics of chronic conditions.^1^ The antiretroviral therapy (ART) programme for people with HIV is the largest in the world, and the epidemic of tuberculosis (TB) is closely related.^2^ At the same time, there is a growing problem of non-communicable diseases, particularly diabetes, hypertension, asthma, chronic obstructive pulmonary disease and post-TB structural lung damage.^1^ Diabetes is now the leading cause of death for women and affects 1 in 4 South Africans over the age of 45 years.^3,4^ Human immunodeficiency virus on the other hand mainly affects younger people in the age range 20–35 years.^2^ This means that millions of South Africans require chronic medication, often for life, while at the same time being part of the working-age population.

The primary health care services are struggling to cope with the increasing patient numbers and are themselves often less than ideal,^5^ with poor infrastructure, limited human resources, unreliable supply chains and paper-based health information systems. Patients experience long waiting times to obtain medication, have to miss work and lose income, while facilities are often congested by patients that are stable and only need medication.^6^ This unacceptable experience may lead patients to avoid collecting their medication, which contributes to poor adherence and control of chronic diseases. As a result of these challenges, particularly in the HIV programme, the National Department of Health supported the development of alternative mechanisms for delivering medication to stable patients with chronic diseases.^6^

The current epidemic of the coronavirus disease 2019 (COVID-19) led to the re-organisation of primary health care services in the Cape Town Metro Health Services (MHS).^7^ One of the innovations within this re-organisation was the use of community health workers (CHWs) to deliver medication at home in order to decongest facilities and protect people with chronic conditions from COVID-19.^8^ As the COVID-19 epidemic began to subside the MHS considered whether to sustain this intervention and commissioned an evaluation of home deliveries by CHWs. In addition, they wanted a rapid review of alternative approaches to delivery of medication in South Africa to guide future policy.

The aim of this study was to conduct a scoping review of alternative mechanisms for delivery of medication to patients within the South African primary health care setting. In particular, the review addressed the following questions:

- What alternative mechanisms have been attempted in South Africa to deliver chronic medication to patients in primary care?
- What do we know about the implementation of these alternative mechanisms?
- What is the effect of these alternative mechanisms on patients, service delivery and clinical outcomes?

## Methods

### Study design

This was a scoping review of published and grey literature in South Africa to address the three questions outlined above.

### Search strategy

The search looked at English articles published in the last 10-years (2010–2020) in PubMed, Google Scholar and Sabinet. The following search terms were used: South Africa, medication systems, primary health care, primary *care, medication adherence, chronic disease, decongest* and *congest, and home delivery of medication. These terms were combined in a number of search strings as shown in Box 1. Delivery was defined as the mechanism by which medication is delivered to or collected by the patient after it has been dispensed by a pharmacist according to the prescription. Any patient related effects, service delivery or clinical outcomes were included, and there was no exclusion of articles on this basis.

BOX 1 Search strings used in scoping review.

“South Africa” AND “medication systems” AND “primary health care”

“South Africa” AND “medication systems”

“South Africa” AND “chronic disease” AND “medication adherence” AND “primary *care”

“South Africa” AND “chronic disease” AND “medication adherence”

“South Africa” AND “chronic disease” AND “*congest” AND “primary *care”

“South Africa” AND “chronic disease” AND “*congest”

“South Africa” AND “chronic disease” AND “decongest*” AND “primary *care”

“South Africa” AND “chronic disease” AND “decongest*”

“South Africa” AND “home delivery of medication” AND “primary *care”

“South Africa” AND “home delivery of medication”

Relevant articles were selected using first the title and then the abstract from each search. Duplicate articles were then removed and the full text of the remaining articles was obtained. Relevant articles were included or excluded after reading the full text. Two researchers performed the searches and any uncertainty about including or excluding an article was resolved through discussion. The reference lists of included full text articles were also examined for any additional eligible literature.

Projects to deliver medication via alternative means in primary health care, which were not identified in the published literature, were also looked for via Google, key individuals at academic departments of family medicine, the department of health, funders such as the Bill and Melinda Gates Foundation, and consultancies such as Percept. Additional initiatives were approached for any unpublished reports or information on websites that could be included in the review.

### Extraction of data

Data was extracted into a standardised Microsoft Excel template according to the following fields:

- Authors.
- Year of publication.
- Location of study or initiative.
- Aim/purpose of the publication.
- Type of publication (e.g. original research, report).
- Methods (if relevant, a summary of the methods used).
- Description of the medication delivery mechanism/model.
- Goals of the delivery mechanism.
- Evidence on the feasibility/fidelity of implementation (key influencing factors, modifications to the design to make it work).
- Evidence on the cost of the delivery mechanism.
- Evidence on the coverage or scalability of the delivery mechanism.
- Evidence on the effect or effectiveness of the delivery mechanism.
- Limitations of the work as reported by the authors.

### Data analysis

The extracted data was collated in an Excel sheet template where all data from each field could be reviewed in a single column. The characteristics (e.g. year of publication, location, type of article, study design) of published articles were analysed quantitatively and reported as frequencies. The other data was analysed qualitatively in order to address the study questions. Key themes in the data were identified and reported in text or tables.

## Findings

**FIGURE 1:**
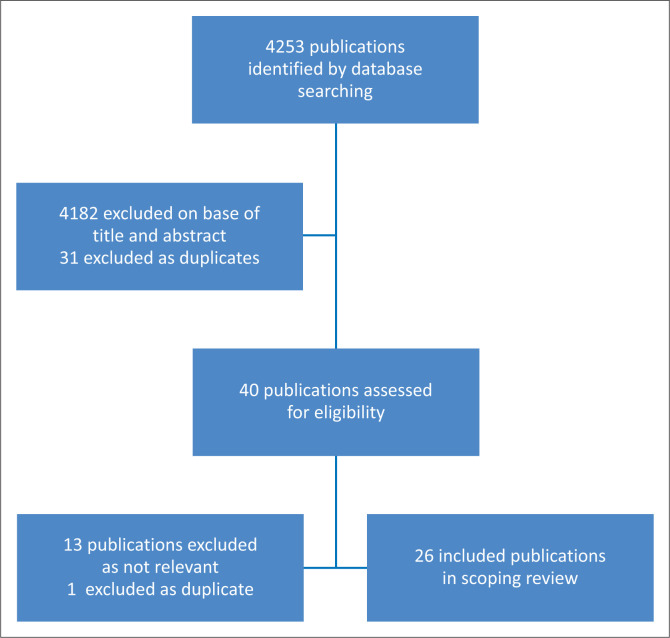
Flow diagram for selection of publications.

The search strategies yielded 4253 publications, and after screening by title and abstract 71 were relevant to the review (Figure 1). Once duplicates (31) were removed, the remaining 40 full text publications were assessed and a further 14 (13 found to be irrelevant and 1 being duplicate) were excluded, leaving 26 in the review.

### Characteristics of the included articles

Altogether, 26 published articles were included in the review (Table 1). Most of the 26 articles were published in the last 5-years and came from the Western Cape (Table 2). A number of studies were also conducted in Gauteng, KwaZulu-Natal, Limpopo and North West. A substantial number (*n* = 17) focused on HIV and evaluated the use of adherence clubs. The most common type of study was qualitative, descriptive and exploratory using interviews with key informants or mixed methods. There was however quite a range of methods that also included experimental, observational and action research studies.

**TABLE 1:** Studies included in the scoping review.

Article	First author	Year	Location	Aim/Purpose of publication	Publication type	Study design	Model type	Disease focus
1	Brey et al.^8^	2020	Western Cape	To describe the home delivery of medication by CHWs during the Coronavirus pandemic	Short report	Not applicable	Home delivery	All chronic diseases
2	Dorward et al.^9^	2020	KwaZulu-Natal	To explore how CCMDD influences engagement in HIV care	Original research	Qualitative exploratory descriptive	Alternative PuPs	HIV
3	Gausi.^10^	2020	Western Cape	To assess clinical outcomes in multi-morbid patients with HIV and diabetes/hypertension after 12 months of care through integrated adherence clubs at two primary care clinics in Cape Town	Thesis (Master)	Experimental before and after	Adherence clubs	HIV, NCD
4	Louw et al.^11^	2020	Gauteng	To describe the home delivery of medication by CHWs during the Coronavirus pandemic	Original research	Action research	Home delivery	All chronic diseases
5	Pascoe et al.^12^	2020	Gauteng, KwaZulu-Natal, Limpopo, North West	To describe the strengths and challenges of implementing the Adherence Guidelines for Chronic Diseases as experienced by health providers, patients, and implementing partners	Original research	Qualitative exploratory descriptive	Adherence clubs and Alternative PuPs	HIV
6	Bock et al.^13^	2019	Western Cape	To report on the clinical outcomes among ART clients attending Community Based Adherence Clubs vs fast lane in the primary care facility	Original research	Observational – retrospective descriptive cohort analysis	Adherence clubs vs facility fast lane	HIV
7	Duffy et al.^14^	2019	Gauteng, KwaZulu-Natal, Eastern Cape, Western Cape	To describe enablers, barriers, and benefits of differentiated facility-based and community-based ART treatment distribution models	Original research	Qualitative exploratory descriptive	Facility-based: adherence clubs vs fast lane vs private GP practice. Community-based: adherence clubs vs workplace outreach vs alternative PuPs	HIV
8	Fox et al.^15^	2019	Gauteng, Northwest, Limpopo, KwaZulu-Natal	To evaluate the effectiveness of differentiated ART delivery models in South African facilities (not all arms focused on medication delivery)	Original research	Cluster randomised controlled trial	Adherence clubs vs alternative PuPs	HIV
9	Fox et al.^16^	2018	Gauteng, Northwest, Limpopo, KwaZulu-Natal	To describe the study protocol for a clinical trial to evaluate differentiated ART delivery models in South African facilities	Protocol	Cluster randomised controlled trial	Adherence clubs vs alternative PuPs	HIV
10	Maharaj.^17^	2018	KwaZulu-Natal	To assess the current role of healthcare workers in the CCMDD programme and their challenges	Thesis	Observational cross sectional descriptive	Alternative PuPs	All chronic diseases
11	MacGregor et al.^18^	2018	Western Cape	To assess the health outcomes of patients accessing adherence clubs and to explore the challenges associated with taking them to scale	Original research	Convergent mixed methods	Adherence clubs	HIV
12	Mukumbang et al.^19^	2018	Western Cape	To evaluate the adherence club intervention by a theory-driven realist evaluation approach	Original research	Convergent mixed methods	Adherence clubs	HIV
13	Jagaroo.^20^	2017	South Africa (excluding Western Cape)	To assess the efficacy of the CCMDD programme in preventing stock-outs in public healthcare in South Africa	Thesis	Convergent mixed methods	Alternative PuPs	All chronic diseases
14	Khuzwayo et al.^21^	2017	KwaZulu-Natal	To explore community awareness and perceptions with regard to ward-based outreach teams	Original research	Qualitative exploratory descriptive	Home delivery	All chronic diseases
15	Magadzire et al.^22^	2017	Western Cape	To evaluate reasons for missed appointments to collect medication linked to the CDU and the implications for loss-to-follow up	Original research	Exploratory mixed methods	Alternative PuPs	All chronic diseases
16	Tsondai et al.^23^	2017	Western Cape	To report clinical outcomes of adherence club patients during scale up	Original research	Observational cross sectional analytical	Adherence clubs	HIV
17	Bango et al.^24^	2016	Western Cape	To assess the cost-effectiveness of lay health worker-led adherence clubs vs. usual care	Original research	Cost-effectiveness analysis	Adherence clubs	HIV
18	Fraser et al.^25^	2016	Gauteng, North West, Limpopo, KwaZulu-Natal	To evaluate the National Adherence Guidelines for Chronic Diseases in South Africa using routinely collected data	Enrolment report	Observational cohorts	Adherence clubs vs alternative PuPs	HIV
19	Magadzire et al.^26^	2016	Western Cape	To provide a typology of community-based distribution models and outline perceived facilitators and barriers to their implementation.	Original research	Qualitative exploratory descriptive	Alternative PuPs and home delivery	All chronic diseases
20	Tshuma et al.^27^	2016	Gauteng and Mpumalanga	To establish the perceived challenges of moving adherence clubs from health facilities to communities	Original research	Qualitative exploratory descriptive	Adherence clubs	HIV
21	Wilkinson.^28^	2016	Western Cape	To describe the scale up of adherence clubs across the Metro Health Services	Original research	Observational cross sectional descriptive	Adherence clubs	HIV
22	Magadzire et al.^29^	2015	Western Cape	To explore the effect of the chronic dispensing unit on improved access to medicines	Original research	Qualitative case study	Alternative PuPs	All chronic diseases
23	Bemelmans et al.^30^	2014	Western Cape	To describe a community-supported model of ART delivery to manage stable patients with HIV	Original research	Review	Adherence clubs	HIV
24	Luque-Fernandez et al.^31^	2013	Western Cape	To evaluate the effectiveness of adherence clubs vs. usual care in maintaining or improving long-term retention-in-care and virologic suppression	Original research	Observational analytical	Adherence clubs	HIV
25	Ndou et al.^32^	2013	Gauteng	To rapidly assess a CHW pilot programme to improve the management of hypertension and diabetes	Original research	Convergent mixed methods	Home delivery	NCDs
26	Wilkinson.^33^	2013	Western Cape	To describe the ART adherence club model	Short report	Not applicable	Adherence clubs	HIV

CCMDD, Central Chronic Medicines Dispensing and Distribution; NCD, non-communicable diseases such as diabetes and hypertension; HIV, human immunodeficiency virus; ART, antiretroviral treatment; PuP, pick-up-point; CDU, Central Dispensing Unit; CHW, community health workers.

**TABLE 2:** Characteristics of included articles (*N* = 26).

Characteristic	*n*
**Year of publication**	
2011–2012	0
2013	3
2014	1
2015	1
2016	5
2017	4
2018	4
2019	3
2020	5
**Location of publication**	
Western Cape	15
Gauteng	9
KwaZulu-Natal	9
Eastern Cape	2
Limpopo	5
North West	5
Mpumalanga	2
Northern Cape	1
Free State	1
**Type of article**	
Mixed methods	5
Cost-effectiveness	1
Observational cross-sectional descriptive	2
Observational cross-sectional analytical	2
Observational cohort	1
Experimental before-and-after	1
Experimental randomised controlled trial	2
Qualitative exploratory descriptive	6
Qualitative case study	1
Action research	1
Protocol	1
Short report	2
Review	1
**Type of delivery system**	
Alternative pick-up points	14
Adherence or support clubs	17
Home delivery	5
Workplace outreach	1
**Type of chronic condition**	
All chronic conditions	8
HIV	17
Non-communicable diseases	2

HIV, human immunodeficiency virus.

### Typology and description of alternative mechanisms

Table 3 outlines the different alternative mechanisms. In all these approaches, criteria are used to select stable patients with chronic conditions who do not have to be seen more frequently by a clinician. How stable is defined will depend on the condition, but in HIV, for example, it typically means someone who had been on ART for at least 12 months, has a current cluster of differentiation 4 (CD4) count > 200 cells/mL, and is virologically suppressed.^9^ Medication must also be pre-packaged, and this can be done centrally by a dispensing unit or by the pharmacy at the local primary care facility. Going to scale requires a high-volume centralised system of pre-packaging the medication.

**TABLE 3:** Description of alternative delivery mechanisms.

Type of delivery mechanism	Description
Alternative PuP	Patients collect pre-packaged medication from a more convenient PuP. A variety of PuP is used such as community halls, schools, private general practitioners, private pharmacies or fast-lanes at the primary care facility. Medication may be pre-packaged by a central dispensing unit. The largest example of this mechanism is the Central Chronic Medicines Dispensing and Distribution (CCMDD) programme which operates in eight provinces in South Africa. An equivalent mechanism, the Central Dispensing Unit (CDU) operates in the Western Cape only. In some projects, medication was pre-packaged by the facility’s pharmacy.
Workplace outreach	The primary care facility performed an outreach clinical service to remote farming communities, which included the delivery of chronic medication.
Adherence or support clubs	A group of 15–30 selected, stable ART patients meet as a club for 30–60 min every 2–3 months at the primary care facility or community venue such as an NGO (non-government organisation), church or mosque. The group is usually facilitated by a CHW, lay health worker or sometimes a nurse. Patients collect their pre-packaged medication at the group and can also receive health education, screening for problems, adherence and peer support. There are typically five club visits per year. Patients attend the primary care facility for a check-up every 6 months or have the routine examination and laboratory tests integrated into their club visits. Problems can be identified and patients are referred back to the primary care facility if necessary. Patients may be allowed to send a buddy on alternate visits to collect on their behalf. Patients must continue to meet the criteria for adherence or control to remain as club members.
Home delivery	A pre-packaged medication parcel is delivered to the patient’s home by a CHW or local entrepreneur. CHWs are part of the health system and can also perform other activities during the visit. They can refer patients with problems to the primary care facility. Parcels must be organised by geographic areas corresponding to the CHW teams. CHW team leaders collect medication from the pharmacy or have it delivered to a local PuP. Local entrepreneurs pick up medication from the pharmacy at the primary care facility and deliver the medication for a fee.
Smart lockers^34^	Internet enabled lockers that can be placed at convenient PuPs such as clinics, shopping malls, pharmacies or commuter nodes. Patients can open their locker with their cell phone and a one-time-password in order to collect their medication.
Pharmacy dispensing units^35^	ATM-like interface with robotic technology that dispenses medication on request with cloud technology to enable remote dispensing, labelling and consultation with a pharmacist.

ART, antiretroviral therapy; CHWs, community health workers; PuP, pick-up-point; ATM, automated teller machine.

Smart lockers and pharmacy dispensing units have been piloted in Gauteng, but no published studies or scientific papers evaluating the innovations were found.^34,35^ Smart lockers are marketed under the name Pelebox and automated teller-like machines (ATM) are trademarked as pharmacy dispensing units.

Regardless of the system the goals were very similar. Goals included:

- Decongesting the facility and reducing the workload so that more attention can be given to new, unstable or sick patients. Specially in 2020, to also reduce the risk of exposure to COVID-19.
- Task-shifting to CHWs in order to address human resource shortages at the primary care facility.
- Providing a more efficient, convenient and patient-centred system for those who are doing well and just need medication. Reducing the burden on patients in terms of travel, costs and time spent at primary care facilities.
- To improve retention in care, adherence to medication and therefore clinical outcomes.
- To improve access to chronic medication and reduce stock-outs by having a more predictable demand that can be planned for.

### Implementation of alternative mechanisms

Table 4 compares the different mechanisms in terms of the feasibility of implementation, scalability and cost. Smart lockers and workplace outreach were excluded as there was insufficient evidence. Several issues are cross-cutting across all the alternative mechanisms:

- The mechanism must be supported by clear unambiguous policy, guidelines or standardised operating procedures and consistent leadership.
- There must be a steady supply of medication with minimal out-of-stock items with procurement linked to quantified demand from registered patients.
- Patients must be motivated and educated on how the system works, and supported to change their behaviour.
- Health services must understand patient or health service related barriers that prevent people from effectively utilising these alternative systems.
- Facility- and community-based health workers must be trained on how to use the system (e.g. selection criteria, scripting).
- There must be an electronic communication system to inform patients on key issues (e.g. date of pick up or delivery, venue) and allow patients to ask questions.
- There must be a reliable electronic audit trail to monitor the safety, status and location of the parcel.
- Complex systems require good communication and feedback between all role players such as private sector centralised dispensing units, district management, facility pharmacists and CHW teams.
- There must be a mechanism to link patients back into care if they develop a problem and to ensure they attend the 6-monthly review.
- Consideration must be given to issues of inadvertent disclosure with stigma or discrimination, particularly for those who are HIV-positive.

A number of more specific issues around the feasibility of implementation are listed in Table 4. All mechanisms have the possibility of going to scale; although the feasibility of this will vary by context. Furthermore, clubs and home delivery have opportunity costs for health workers who if they support the system for alternative delivery will not then be able to perform other duties in the time available. Central Chronic Medicines Dispensing and Distribution has gone to scale nationally and is a flagship project of the National Department of Health, combining centralised dispensing and alternative PuPs. Pharmacy dispensing units have been piloted but remain underutilised. They may be more efficient if linked to a centralised medication supply rather than a facility.

**TABLE 4:** Feasibility, scalability and cost of different mechanisms.

Mechanisms	Feasibility	Scalability	Cost
Alternative PuPs	Inflexible dates or times for pick up Restrictions on types of medication (e.g. TB prophylaxis) Too rigid enrolment criteria exclude most vulnerable	By August 2020 CCMDD had 2 592 513 active registered patients (National Department of Health dashboard) Centralised automated dispensing can support thousands of alternative PuPs and 60% of patients with chronic conditions In Western Cape, non-pick-up rate of 8% – 12%	No data published on actual costs
Adherence clubs	Mistrust and confidentiality amongst group members Staff motivation important to establish and maintain clubs Need suitable community venues and space	The number of clubs can increase to scale, but require dedicated coordination from the facility and sufficient professional staff for clinical activities at the clubs	Costs per patient per year estimated (2014–2016) between R438.00 – R2268.00 for clubs vs R824.00 – R2824.00 for standard care
Home delivery^36^	Need accurate addresses and contact numbers Need transport to deliver medication to CHW teams Need to resource CHWs and protect them from crime Patient or delegated person must be at home to receive parcel Out-of-area patients are a challenge CHWs need support and coordination from nurse supervisors and with facilities Entrepreneurs have no training in health	820 000 parcels delivered over 6-months in Cape Town and once implemented 81% of pre-packaged medication was delivered with a 9% return rate CHWs must balance time spent on home delivery vs other roles	Additional cost (2020) R15.00 per patient delivery (R178.00 per patient year). Opportunity costs for staff time (2020) R39.00 per patient delivery Entrepreneurs charge R10.00 – R20.00 per delivery (2020)
Pharmacy Dispensing Unit^37^	Patient support needed initially to use the PDU	91% collection rate and 9% non-collection that requires follow up Between 15 000–20 000 dispenses a month from 18 PDUs operating at only 10.5% capacity	High set-up costs (2018), R8.35 million per site and R3 50 000.00 per month running costs with capacity for 22 000 dispenses Cost per dispense at low utilisation (2018) is R268.00 and could drop to R67.00 with high utilisation versus clinic at R110.00 versus R59.00 at adherence club

TB, tuberculosis; CCMDD, Central Chronic Medicines Dispensing and Distribution; PDU, pharmacy dispensing unit; CHWs, community health workers; PuP, pick-up-point.

### Cost and effectiveness of alternative mechanisms

These alternative mechanisms had many benefits for patients and were positively received. Patients saved time and money in not having to attend the primary care facility; and obtaining medication was less disruptive to work and other responsibilities.^14,15,27^ Attending the facility implied long waiting times during restrictive opening hours as well as the cost of transport and lost income.^19^

Facilities reported a decrease in congestion, reduced workload and alleviation of staff shortages.^27^ Stock-outs were less frequent with the alternative PuPs because the quantification of patients on different items was improved and procurement was more effective in a centralised dispensing system.^20^

Most mechanisms demonstrated improved adherence in terms of collecting monthly medication and for HIV this also translated into improved retention in care. At 40-months there was 97% retention in adherence clubs vs 85% in usual care for similarly eligible patients.^33^ There was some evidence that retention in care was higher in men, but less in younger patients (< 25 years) and in facilities with larger cohorts of patients.^15^ Home deliveries were also associated with improved retention in care with more patients receiving their medication (8.9% non-delivery vs 12% non-collection at facilities).^36^ Forty-two percent of patients on home delivery also reported taking their medication more often.^36^ In addition, adherence clubs showed a follow through to viral suppression (67% improvement in club vs usual clinic care).^30^

Although we only had one cost-effectiveness study on one mechanism,^24^ less robust evidence alludes to alternative mechanisms being cheaper and more beneficial than attending the facility to collect medication.

Pharmacy dispensing units have high start-up costs and are not yet cost-effective because of underutilisation.^35^ There is a need for more comprehensive economic evaluations of different systems, particularly at scale.

## Discussion

Alternative PuPs and adherence clubs have already been widely implemented in South Africa with support from the National Department of Health. The COVID-19 pandemic put home delivery of medication in the spotlight as it was an appropriate response to decongesting facilities and national lockdown. Home delivery may be through CHWs in Ward Based Primary Health Care Outreach Teams or in some areas by local entrepreneurs.^38^ Going to scale requires a chronic dispensing unit to pre-package medication centrally. Smart lockers and pharmacy dispensing units are more recent technologically driven mechanisms that have been piloted on a smaller scale.

The goals of these alternative delivery systems were broadly similar and the need for them was driven by the HIV epidemic and unprecedented numbers of people on long-term ARTs that were overwhelming health facilities. Decongesting facilities to allow the available health professionals to focus on new and unstable patients was a central goal. Access to medication was also limited by the unacceptable waiting times at facilities and it was hoped that alternative mechanisms would improve access, adherence and ultimately clinical outcomes. The growing prevalence of non-communicable chronic diseases has highlighted the need to extend these alternative mechanisms to all people with chronic conditions. This need was further emphasised by the COVID-19 pandemic where HIV and diabetes were both significant risk factors for severe disease.^39^

Patients were generally very positive about these alternative mechanisms once they learnt to trust the new systems. They substantially reduce the burden of collecting medication, saving both time and money, while decongesting facilities and capacitating health workers. These initiatives can be seen as supporting key aspects of effective primary health care,^40^ particularly accessibility and to some extent the person-centredness of services. There is good evidence that they improve collection of medication and therefore adherence and retention in care, but very limited evidence of the effect on clinical outcomes.

Although the cost per dispense is relatively low and better than the cost of dispensing at the facility level,^19,24,30^ the costs aggregate to substantial amounts when taken to scale. For example, the cost of home delivery of medication by CHWs in Cape Town could be 1.3% of total expenditure for Metro Health Services if sustained (R3 800 000.00 in 2020), although given the potential benefits this might be regarded as cost-effective.^36^ The national cost of CCMDD (with a hybrid of alternative PuPs, adherence clubs and fast-lanes) has been predicted (2016) as between R1.5 billion and R3bn per year with 9.1 million patients registered.^41^ At this point the start-up and ongoing costs of PDUs are less competitive, and there is little data available on smart lockers.

The review only looked at literature from South Africa to limit its scope and to enable a rapid, but relevant review process. The combination of databases should have identified articles in relatively high impact journals (PubMed), articles in lower impact journals not in PubMed (Google Scholar) as well as studies published on local university and institutional websites (Sabinet), although databases such as Scopus were not included. It is likely therefore that all relevant articles were included, particularly as effort was also made to obtain grey literature and study the references of included articles.

The evidence cannot support definitive recommendations on what combination of mechanisms will be most cost-effective, but does support the conclusion that these alternative systems mostly achieved their goals. Each mechanism has a number of pros and cons, and may suit different types of patients and different regional contexts throughout the country. A hybrid system therefore that offers a range of alternatives to match the needs of different patients seems ideal, while avoiding unmanageable complexity. Such a system might include:

- Alternative PuPs for the majority of patients at locations that are convenient and accessible, particularly for working people. Consider installing smart lockers at key locations that are accessible 24-h a day. PuPs in the community rather than fast-lanes in facilities may decongest facilities more effectively.
- Adherence or support clubs for a limited number of patients that can be managed by the available human resources at a given facility, particularly for those that need additional support, for example, more isolated, recently diagnosed or borderline in terms of control and stability.
- Home delivery of medication by CHWs, particularly for those that are frail, disabled or housebound. Ensure that CHWs have sufficient capacity to still perform a comprehensive service. Home delivery may also suit working people who pay a local entrepreneur to deliver at home.

To remain person-centred there should be a process of mutual decision-making, with health workers identifying eligible patients, but negotiating which option will work best for them. Being respectful of patient choice and control is likely to improve patient utilisation and satisfaction.^42^ The whole system should be supported by a centralised dispensing unit that automates the packaging and dispensing of medication in order to manage high volumes.

## Conclusion

Six alternative medication delivery systems were identified in South Africa: alternative PuPs, adherence clubs, workplace outreach, home delivery, smart lockers, and automated pharmacy dispensing units. Alternative PuPs and adherence clubs combined with centralised dispensing units have already been widely implemented. Home delivery was emphasised as an appropriate option in response to the COVID-19 pandemic. More technologically driven options have been piloted. All of these options appear to improve accessibility of medication and are acceptable to patients with the likelihood of improved adherence, retention in care and possibility of improved clinical outcomes. A hybrid system that offers a manageable range of options to eligible patients in a person-centred approach is probably the way forward. Further research is needed into the most cost-effective combination of delivery systems to meet the diverse needs of patients as well as the effect on clinical outcomes.

## References

[1] Bradshaw D, Norman R, Schneider M. A clarion call for action based on refined DALY estimates for South Africa. S Afr Med J. 2007;97:438–440. 17691472

[2] Allinder S. The world’s largest HIV epidemic in crisis: HIV in South Africa [homepage on the Internet]. Center for Strategic and International Studies; 2019 [cited 2020 Nov 05]. Available from: https://www.csis.org/analysis/worlds-largest-hiv-epidemic-crisis-hiv-south-africa#

[3] The Society for Endocrinology M and D of SAT 2 DGEC. SEMDSA guidelines for the management of type 2 diabetes. JEMDSA. 2017;22:S1–S196.

[4] StatsSA. Key find. P0309.3 – Mortal causes death South Africa Findings from death Notification, 2015 [homepage on the Internet]. 2018 [cited 2019 Mar 26]. Available from: http://www.statssa.gov.za/?page_id=1856&PPN=P0309.3&SCH=6987

[5] Hunter J, Chandran T, Asmall S, et al. The ideal clinic in South Africa: Progress and challenges in implementation. In: Padarath A, Barron P, eds. South African Health Review. 20th ed. Cape Town: Health System Trust, 2017: p. 111–124.

[6] CCMDD. Get checked go collect, free chronic medication [homepage on the Internet]. [cited 2019 May 19]. Available from: https://getcheckedgocollect.org.za/ccmdd/

[7] Mash R, Goliath C, Perez G. Re-organising primary health care to respond to the Coronavirus epidemic in Cape Town, South Africa. Afr J Prim Health Care Fam Med. 2020;12(1):4. 10.4102/phcfm.v12i1.2607 PMC7669993 33181873

[8] Brey Z, Mash R, Goliath C, Roman D. Home delivery of medication during Coronavirus disease 2019, Cape Town, South Africa: Short report. Afr J Prim Health Care Fam Med. 2020;12(1):2449. 10.4102/phcfm.v12i1.2449 PMC7284162 32501022

[9] Dorward J, Msimango L, Gibbs A, et al. Understanding how community antiretroviral delivery influences engagement in HIV care: A qualitative assessment of the Centralised Chronic Medication Dispensing and Distribution programme in South Africa. BMJ Open. 2020;10(5):35412. 10.1136/bmjopen-2019-035412 PMC7245406 32414827

[10] Gausi B. Assessing the effectiveness of integrated non-communicable disease and antiretroviral adherence clubs in Cape Town, South Africa [homepage on the Internet]. Faculty of Health Sciences; 2020 [cited 2020 Nov 12]. Available from: https://open.uct.ac.za/handle/11427/32219

[11] Louw JM, Rantloane B, Ngcobo S, et al. Home delivery of medication as part of reducing congestion in primary healthcare in Tshwane District Health Services. South African J Public Health. 2020;4(2):50.

[12] Pascoe SJS, Scott NA, Fong RM, et al. ‘Patients are not the same, so we cannot treat them the same’ – A qualitative content analysis of provider, patient and implementer perspectives on differentiated service delivery models for HIV treatment in South Africa. J Int AIDS Soc. 2020;23(6):e25544. 10.1002/jia2.25544 32585077 PMC7316408

[13] Bock P, Gunst C, Maschilla L, et al. Retention in care and factors critical for effectively implementing antiretroviral adherence clubs in a rural district in South Africa. J Int AIDS Soc. 2019;22(10):e25396. 10.1002/jia2.25396 31588668 PMC6778813

[14] Duffy M, Sharer M, Davis N, et al. Differentiated antiretroviral therapy distribution models: Enablers and barriers to universal HIV treatment in South Africa, Uganda, and Zimbabwe. J Assoc Nurses AIDS Care. 2019;30(5):E132–E143. 31135515 10.1097/JNC.0000000000000097 PMC6756295

[15] Fox MP, Pascoe S, Huber AN, et al. Adherence clubs and decentralized medication delivery to support patient retention and sustained viral suppression in care: Results from a cluster-randomized evaluation of differentiated ART delivery models in South Africa. PLoS Med. 2019;16(7):e1002874. 31335865 10.1371/journal.pmed.1002874 PMC6650049

[16] Fox MP, Pascoe SJ, Huber AN, et al. Assessing the impact of the National Department of Health’s national adherence guidelines for chronic diseases in South Africa using routinely collected data: A cluster-randomised evaluation. BMJ Open. 2018;8(1):19680. 10.1136/bmjopen-2017-019680 PMC5781226 29358446

[17] Maharaj ML. Assessment of factors affecting Healthcare workers involved in the Centralised Chronic Medicines Dispensing and Distribution (CCMDD) programme: The case of eThekwini Metropolitan Health district, South Africa [homepage on the Internet]. 2018 [cited 2020 Nov 12]. Available from: https://researchspace.ukzn.ac.za/handle/10413/17809

[18] MacGregor H, McKenzie A, Jacobs T, Ullauri A. Scaling up ART adherence clubs in the public sector health system in the Western Cape, South Africa: A study of the institutionalisation of a pilot innovation. Global Health. 2018;14:40. 29695268 10.1186/s12992-018-0351-z PMC5918532

[19] Mukumbang FC, Marchal B, Van Belle S, Van Wyk B. ‘Patients are not following the [adherence] club rules anymore’: A realist case study of the antiretroviral treatment Adherence Club, South Africa. Qual Health Res. 2018;28(12):1839–1857. 30033857 10.1177/1049732318784883 PMC6154254

[20] Jagaroo N. Efficacy of the central chronic medicine dispensing and delivery programme in South Africa [homepage on the Internet]. 2017 [cited 2020 Oct 26]. Available from: https://hdl.handle.net/10539/27675

[21] Khuzwayo LS, Moshabela M. The perceived role of ward-based primary healthcare outreach teams in rural KwaZulu-Natal, South Africa. Afr J Prim Health Care Fam Med. 2017;9(1):1388. 10.4102/phcfm.v9i1.1388 PMC5458574 28582992

[22] Magadzire BP, Mathole T, Ward K. Reasons for missed appointments linked to a public-sector intervention targeting patients with stable chronic conditions in South Africa: Results from in-depth interviews and a retrospective review of medical records. BMC Fam Pract. 2017;18:82. 28836941 10.1186/s12875-017-0655-8 PMC5571491

[23] Tsondai PR, Wilkinson LS, Grimsrud A, Mdlalo PT, Ullauri A, Boulle A. High rates of retention and viral suppression in the scale-up of antiretroviral therapy adherence clubs in Cape Town, South Africa. J Int AIDS Soc. 2017;20(S4):21649. 10.7448/IAS.20.5.21649 28770595 PMC5577696

[24] Bango F, Ashmore J, Wilkinson L, Van Cutsem G, Cleary S. Adherence clubs for long-term provision of antiretroviral therapy: Cost-effectiveness and access analysis from Khayelitsha, South Africa. Trop Med Int Heal. 2016;21(9):1115–1123. 10.1111/tmi.12736 27300077

[25] Fraser N, Shubber Z, Gorgens M. Evaluation of the national adherence guidelines for chronic diseases in South Africa: Healthcare provider perspectives on different care models, 2017 [homepage on the Internet]. 2017 [cited 2020 Nov 12]. Available from: http://akb.africa-union.org/auc/handle/AKB/11117

[26] Magadzire BP, Marchal B, Ward K. Novel models to improve access to medicines for chronic diseases in South Africa: An analysis of stakeholder perspectives on community-based distribution models. J Pharm Policy Pract. 2016;9:28. 10.1186/s40545-016-0082-6 27733918 PMC5045655

[27] Tshuma N, Mosikare O, Yun JA, et al. Acceptability of community-based adherence clubs among health facility staff in South Africa: A qualitative study. Patient Prefer Adherence. 2017;11:1523–1531. 28979100 10.2147/PPA.S116826 PMC5602677

[28] Wilkinson L, Harley B, Sharp J, et al. Expansion of the Adherence Club model for stable antiretroviral therapy patients in the Cape Metro, South Africa 2011–2015. Trop Med Int Heal. 2016;21(6):743–749. 10.1111/tmi.12699 27097834

[29] Magadzire BP, Marchal B, Ward K. Improving access to medicines through centralised dispensing in the public sector: A case study of the Chronic Dispensing Unit in the Western Cape Province, South Africa. BMC Health Serv Res. 2015;15:513. 26577831 10.1186/s12913-015-1164-x PMC4650275

[30] Bemelmans M, Baert S, Goemaere E, et al. Community-supported models of care for people on HIV treatment in sub-Saharan Africa. Trop Med Int Heal. 2014;19(8):968–977. 10.1111/tmi.12332 24889337

[31] Luque-Fernandez MA, Van Cutsem G, Goemaere E, et al. Effectiveness of patient adherence groups as a model of care for stable patients on antiretroviral therapy in Khayelitsha, Cape Town, South Africa. PLoS One. 2013;8(2):e56088. 10.1371/journal.pone.0056088 23418518 PMC3571960

[32] Ndou T, Van Zyl G, Hlahane S, Goudge J. A rapid assessment of a community health worker pilot programme to improve the management of hypertension and diabetes in Emfuleni sub-district of Gauteng Province, South Africa. Glob Health Action. 2013;6(1):19228. 23364086 10.3402/gha.v6i0.19228 PMC3556684

[33] Wilkinson LS. ART adherence clubs: A long-term retention strategy for clinically stable patients receiving antiretroviral therapy. South Afr J HIV Med. 2013;14(2):a77.

[34] Technovera. What are smart lockers [homepage on the Internet]. 2021 [cited 2021 Jan 12]. Available from: https://www.pelebox.com/

[35] Rightepharmacy. Pharmacy dispensing unit [homepage on the Internet]. [cited 2021 Jan 13]. Available from: https://rightepharmacy.co.za/solutions/pdu-atm-pharmacy/

[36] Mash R, Schouw D, Daviaud E, Besada D, Roman D. Evaluating the implementation of home delivery of medication by community health workers during the COVID-19 pandemic in Cape Town, South Africa: A convergent mixed methods study. Cape Town: Metro Health Services; 2020. 10.1186/s12913-022-07464-x PMC8784590 35073888

[37] Venter F, Pienaar D. The cost-effectiveness of pharmacy dispensing units client: A comparative evaluation. Pretoria; 2018.

[38] Iyeza. Iyeza health [homepage on the Internet]. [cited 2021 Jan 13]. Available from: https://www.iyezahealth.co.za/

[39] Boulle A, Davies M-A, Hussey H, et al. Risk factors for COVID-19 death in a population cohort study from the Western Cape Province, South Africa. Clin Infect Dis. 2020:ciaa1198. 10.1093/cid/ciaa1198 32860699 PMC7499501

[40] Primary health care performance initiative (PHCPI) [homepage on the Internet]. [cited 2018 Apr 18]. Available from: https://improvingphc.org/

[41] Project Last Mile. Centralised chronic medications distribution and delivery initiative: Business plan [homepage on the Internet]. Northern Virginia; 2016 [cited 2021 Jan 12]. Available from: https://www.projectlastmile.com/

[42] Louw JM, Marcus TS, Hugo J. How to measure person-centred practice – An analysis of reviews of the literature. Afr J Prim Health Care Fam Med. 2020;12(1):a2170. 10.4102/phcfm.v12i1.2170 PMC7136800 32129646

